# Dispersed Activity during Passive Movement in the Globus Pallidus of the 1-Methyl-4-Phenyl-1,2,3,6-Tetrahydropyridine (MPTP)-Treated Primate

**DOI:** 10.1371/journal.pone.0016293

**Published:** 2011-01-18

**Authors:** Yaara Erez, Hadass Tischler, Katya Belelovsky, Izhar Bar-Gad

**Affiliations:** 1 Gonda Brain Research Center, Bar Ilan University, Ramat Gan, Israel; 2 Goodman Faculty of Life Sciences, Bar Ilan University, Ramat Gan, Israel; Johns Hopkins, United States of America

## Abstract

Parkinson's disease is a neurodegenerative disorder manifesting in debilitating motor symptoms. This disorder is characterized by abnormal activity throughout the cortico-basal ganglia loop at both the single neuron and network levels. Previous neurophysiological studies have suggested that the encoding of movement in the parkinsonian state involves correlated activity and synchronized firing patterns. In this study, we used multi-electrode recordings to directly explore the activity of neurons from the globus pallidus of parkinsonian primates during passive limb movements and to determine the extent to which they interact and synchronize. The vast majority (80/103) of the recorded pallidal neurons responded to periodic flexion-extension movements of the elbow. The response pattern was sinusoidal-like and the timing of the peak response of the neurons was uniformly distributed around the movement cycle. The interaction between the neuronal activities was analyzed for 123 simultaneously recorded pairs of neurons. Movement-based signal correlation values were diverse and their mean was not significantly different from zero, demonstrating that the neurons were not activated synchronously in response to movement. Additionally, the difference in the peak responses phase of pairs of neurons was uniformly distributed, showing their independent firing relative to the movement cycle. Our results indicate that despite the widely distributed activity in the globus pallidus of the parkinsonian primate, movement encoding is dispersed and independent rather than correlated and synchronized, thus contradicting current views that posit synchronous activation during Parkinson's disease.

## Introduction

The globus pallidus (GP) plays a key role in the processing of motor, associative and limbic information within the cortico-basal ganglia loop [1]. The GP is divided into an external segment (GPe), an intrinsic nucleus within the basal ganglia (BG), and an internal segment (GPi), a major output nucleus of the BG [2]. The neuronal activity in both segments of the GP is known to be related to the encoding of different movement parameters. In normal primates, pallidal activity is associated with both voluntary (active) and passive movements, and follows a somatotopic organization [3]–[6]. Neuronal activity has also been related to movement velocity [7], direction and amplitude [8] and context [9].

Parkinson's disease (PD) is a neurodegenerative disease manifesting in debilitating motor symptoms. Studies using the 1-methyl-4-phenyl-1,2,3,6-tetrahydropyridine (MPTP) primate model of PD, which replicates the symptoms of the disease, have revealed major changes in the firing patterns of GP neurons. In the parkinsonian state, the neurons undergo a loss of specificity in response to passive movements compared to intact animals. A larger fraction of pallidal cells respond to movement, usually to more than one body part and to multiple joints [10]. Loss of segregation of information has also been found downstream at the GPi targets in the thalamus [11]. Additionally, the independent neuronal firing of the normal GP is replaced by oscillatory correlations between pallidal neurons in the parkinsonian state [12]. Studies on PD patients undergoing stereotaxic surgery have reported similar interactions and firing pattern changes, including the fact that pallidal responses to passive and active movements are partially arranged in somatotopic clusters [13]–[16]. Additionally, there are reports of synchronized neuronal discharge in the GP, but this was limited to oscillatory activity in patients with limb tremor [17].

These converging neurophysiological data, reinforced by current theories of pallidal functionality in the parkinsonian state [18], [19], have shaped current thinking that movement encoding in the BG is correlated and synchronized in PD [20]. The aim of this study was to test this widely held belief by directly exploring the activity of pallidal neurons during passive movements and the extent to which they interact and synchronize in parkinsonian primates.

## Materials and Methods

### Animals and Ethics Statement

Two cynomolgus (*Macaca fascicularis*) male monkeys were used (A-3.7Kg; N-4Kg). The monkeys' water and food consumption and weight were checked daily and their health was monitored by a veterinarian. All procedures were in accordance with the *National Institutes of Health Guide for the Care and Use of Laboratory Animals, Bar-Ilan University Guidelines for the Use and Care of Laboratory Animals in Research* and the recommendations of the *Weatherall Report*. All procedures were approved and supervised by the *Institutional Animal Care and Use Committee (IACUC)*. These procedures were approved by the National Committee for Experiments on Laboratory Animals at the Ministry of Health (permit number BIU150605).

### Surgery and Induction of Parkinsonism

The monkeys underwent a surgical procedure under general anesthesia to attach a recording chamber to the skull allowing access to both segments of the GP. The details of the procedure are described elsewhere [21]. Briefly, a 27 mm square Cilux recording chamber (Alpha-Omega Engineering, Nazareth, Israel) was attached to the right hemisphere for monkey A, and to the left hemisphere for monkey N, with its center targeted at stereotaxic coordinates A12-L7-H14 for monkey A and A13-L8-H13 for monkey N [22]. Parkinsonism was later induced by five intramuscular injections of 0.4 mg/kg 1-methyl-4-phenyl-1,2,3,6-tetrahydropyridine (MPTP)-HCl (Sigma, Rehovot, Israel) given under intramuscular Ketamine-HCl (10 mg/kg) anesthesia over a period of 4 days, after which the monkeys developed severe parkinsonism. The monkeys exhibited all the main parkinsonian symptoms, such as akinesia and rigidity, except for rest tremor which is typically not exhibited by this species. Additionally, both monkeys had dystonia, primarily in the lower limbs. Dystonia was not apparent in the upper limb examined during the recording sessions. The monkeys' parkinsonian state was assessed daily using the Schneider scale [23]. The parkinsonian symptoms were severe and stable throughout the recording period, with almost no voluntary movements or spontaneous activity in any body part (mean±SD: monkey A: 45.75±3.37, monkey N: 46.58±2.71, scale of 0 (asymptomatic) to 53 (maximal symptoms)). Recordings were resumed 4 and 5 days after the last MPTP injection for monkeys A and N, respectively.

### Recording and experimental setup

The monkeys were seated in a primate chair with their head fixed during the recording sessions. Using a cylindrical guide (1.7/2.15 mm inner/outer diameter), eight glass-coated tungsten microelectrodes (impedance 0.2–0.7 MΩ at 1 kHz) were advanced separately (EPS 4.10, Alpha–Omega Engineering, Nazareth, Israel) into the GP. The electrode signal was continuously sampled at 40 kHz (Alphamap 10.10, Alpha–Omega Engineering, Nazareth, Israel), amplified (*1000) and bandpass filtered (2–8000 Hz four-pole Butterworth filter) (MCP-Plus 4.10, Alpha–Omega Engineering, Nazareth, Israel). The distinction between the GPe and internal GPi was determined online based on characteristics of neuronal activity (oscillations, background noise, etc.), and the existence of border cells and white matter fibers between the two segments. Border cells were excluded from this study, and most of the recorded neurons in the GPe were high-frequency pausers.

**Figure 1:**
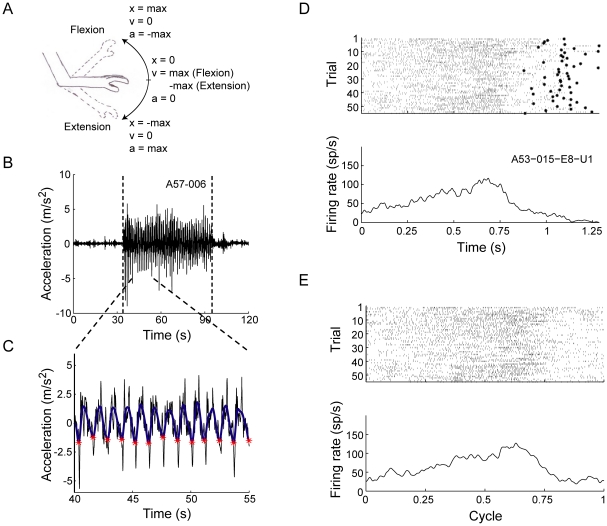
Movement and neuronal response quantification. ***A***, Illustration of the passive movement of the monkey's upper limb, indicating the displacement (x), velocity (v) and acceleration (a) values. ***B***, Example of a raw accelerometer signal. Vertical dashed lines indicate the beginning and end of movement. ***C***, Enlargement of the signal from ***B*** (black), overlaid with the filtered signal (blue) and the identification of the movement cycles (red asterisks). ***D***,***E***, Movement peri-event-time-raster (top) and movement peri-event-time-histogram (mPETH) (bottom) of a GPe cell are presented using (***D***) The original duration of the movement cycles (marked by asterisks) and (***E***) scaled to 1 s cycle duration.

The recording protocol consisted of three consecutive segments: (1) No movement for 30 seconds, which was referred to as baseline (B1); (2) passive upper limb manipulation (M) for 60 seconds; (3) no movement for 30 seconds (B2). An accelerometer (8636C5, Kistler, NY) was attached to the monkeys' contralateral upper limb to monitor movement. The accelerometer was placed near the monkey's wrist and its position and rotation relative to the upper limb were similar throughout the recording days and sessions. Passive movement was used for this study since the monkeys were akinetic and lacked the ability to perform voluntary active movements. Using a stiff surface connected to the monkey's limb from the elbow to the fingers, the wrist joint was fixed with the back of the hand facing upwards, such that movements would involve the elbow joint alone, allowing only one degree of freedom. The movement was applied only when the monkey was seated quietly and was neither resisting nor trying to perform other voluntary movements. The monkey's behavior was monitored by a multi-channel video system (GV-800, Geovision, Taiwan) and was observed by the experimenter who was standing beside the monkey. Limb movement was controlled by the experimenter and was held still or passively moved during the various recording protocol segments. Elbow flexion (upward movement) and extension (downward movement) was applied using the stiff surface in a sinusoidal-like manner, up to an angle of ±30° at a 1 Hz frequency. The sinusoidal-like pattern of movement was chosen since it reflects the acceleration, velocity and displacement representations of movement similarly, as they are all sine or cosine derivatives (Fig. 1A). The accelerometer signal was continuously sampled at 1.25 kHz and was offline filtered (2 Hz six-pole low-pass Butterworth filter) in order to focus on movement frequency rather than on the high frequency artifacts of the measurements. The cycles of movement were later identified based on the offline filtered accelerometer signal (Fig. 1B, C).

### Data analysis

The digitized continuous signal of each electrode was sorted offline (OFS-2.8.4, Plexon, Dallas, TX) to generate one or more spike trains of single units.

#### Single neuron analysis

Mean firing rates before and during movement were calculated by dividing the number of spikes by the recording time, to account for global activation of the neuronal activity. Movement peri-event-time-histograms (mPETHs) aligned to the trough of the acceleration signal were calculated and scaled to a single second cycle, using a 1 ms bin and smoothed by a Gaussian window (SD = 10 ms) (Fig. 1D,E). Each mPETH was fitted using non-linear regression to a one-cycle sine function of the form:
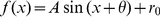
(1)where *A* is the response amplitude (peak-to-mean), *θ* is the phase aligned to the beginning of cycle, and *r_0_* is the mean firing rate during movement. These sine fit parameters are not affected by the single second scaling of the movement cycle. For some cells, a two-cycle sine function was fitted to the mPETH to adjust for the distorted shape of the mPETH due to the rigidity of the monkey and the resulting resistance to movement.

Cells with a large coefficient of determination (*R^2^*≥0.25) and large amplitude modulation (*A*≥5) were considered as significantly responding to movement, and were used for the remainder of the analysis. These threshold parameters were chosen to best describe the data.

#### Interaction analysis

Offset phase (Δθ) was calculated for each pair of neurons, measuring the difference in the peak response phases:
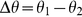
(4)where 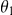 and 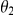 are the phases of the sine fits to the mPETHs of the two neurons. An offset phase close to 0° or 360° implies similar phases of mPETHs of the single neurons, whereas an offset phase close to 180° implies opposite phases.

Additionally, signal and noise correlations were calculated for each pair of simultaneously recorded neurons. This analysis was used to differentiate correlated activity resulting from common response to movement from spontaneous co-activation and thus characterize the network activity and separate it from the activity of single neurons. The correlations were based on the neurons' mPETHs, calculated using a 4 ms bin and smoothed by a Gaussian window (SD = 8 ms).

The signal correlation (SC) measures the correlation between the mean responses of two neurons to movement, representing their tendency to respond similarly, and was defined as:
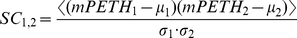
(2)where 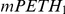 and 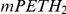 are the mPETHs of the first and second neuron, respectively, 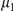 and 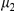 are the means of the two mPETHs, and 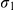 and 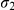 are the standard deviations of the mPETHs.

The noise correlation (NC) measures the inter-cycle firing relations between two neurons, thus quantifying their co-activation independently from their response to movement. The NC was defined as:
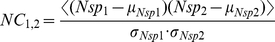
(3)where 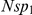 and 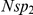 are vectors of the total number of the spikes at each movement cycle for the two neurons, 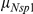 and 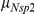 are the mean number of spikes over all cycles for the two neurons, and 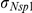 and 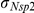 are the standard deviations of the number of spikes over all movement cycles for the two neurons.

Significance tests for SC and NC were calculated using bootstrapping analysis. Surrogate data were generated by randomizing one of the signals (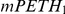 and 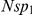, respectively) and calculating the correlation. This process was repeated 500 times, determining the significance based on the ranking of the SC and NC in the surrogate distributions (two-tailed, p<0.01).

All measures are presented as mean±SEM, unless otherwise specified.

### Histology

**Figure 2:**
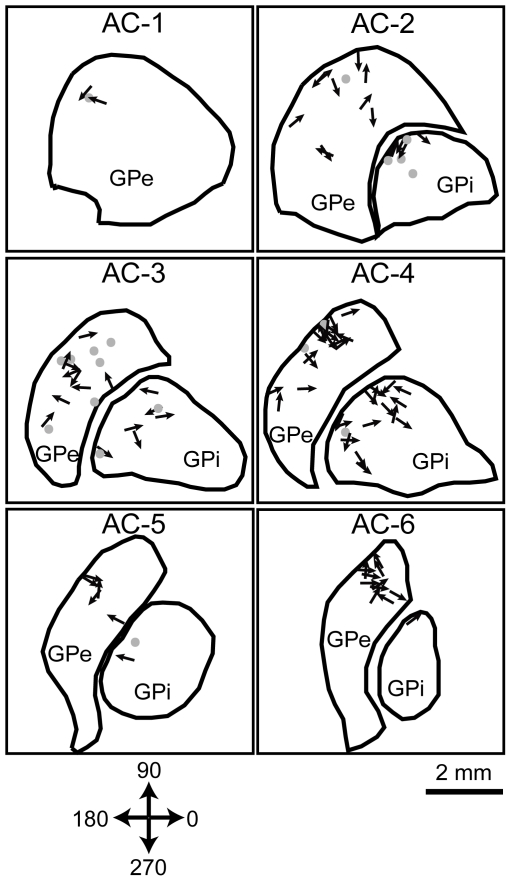
Histological reconstruction of the recording sites. The recording sites, indicated by arrows (movement responding neurons) and circles (non-responding neurons) are superimposed on reconstructed coronal brain slices (monkey N). The direction of each arrow indicates the phase of the peak response relative to the beginning of the movement cycle.

Specifics on the histological procedure are reported elsewhere [21]. Briefly, following the completion of the experiment, the animals were deeply anaesthetized and the brain was removed and cut in the coronal plane using a cryostat (Leica Microsystems, Wetzler, Germany). Each section was digitized using a 10 MPixel digital camera and sections of interest were mounted onto glass slides and Nissl stained. A combination of anatomical and physiological markers was used for the reconstruction of the locations of the recorded sites. The anatomical mapping was based primarily on verification of electrodes tracks in the brain section subsequent to Nissl staining and microlesions solely on monkey N prior to sacrifice. These markers were then merged with the calculated electrode trajectories to reconstruct the recording sites (Fig. 2).

## Results

Pallidal cells from both segments were recorded during sessions combining rest and passive movement periods. A total of 37 sessions were used for this study (26 in monkey A and 11 in monkey N). The mean movement cycle length during these sessions was 1.05±0.02 s. The neuronal activity of the cells was analyzed following offline sorting, and a total of 67 GPe and 36 GPi (70 from monkey A and 33 from monkey N) stable, high-grade cells were used.

### Single neuron analysis

**Figure 3:**
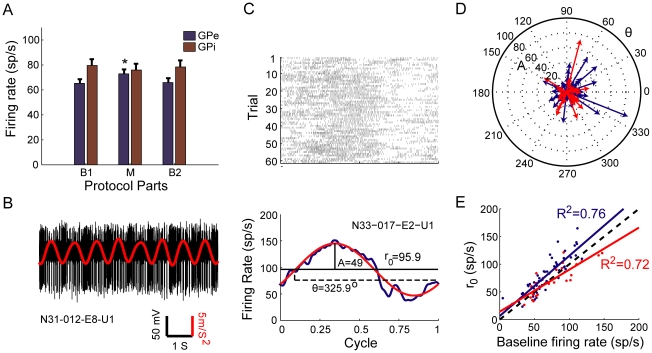
Single neuron movement encoding in the GP. ***A***, Mean firing rates (±SEM) of GPe (blue) and GPi (red) neurons during different parts of the experimental protocol. * p<0.001. ***B***, Example of a raw spike train recorded from a GPi neuron during movement (black). The simultaneously recorded filtered accelerometer signal is superimposed (red). ***C***, Example of a raster (top) and mPETH (bottom, blue) of a GPe cell. The red curve indicates the sine fit for the mPETH, and its parameters are shown (R^2^ = 0.96): horizontal solid line indicates r_0_, horizontal dashed line indicates the phase and vertical line indicates the amplitude. ***D***, Compass plot of the sine fit phase (direction of arrow) and amplitude (length of the arrow) of all analyzed cells. ***E***, Scatter plot of the firing rate prior to movement relative to the r_0_. The regression lines are shown in solid lines, dashed line indicates equality line. In ***D***,***E***, GPe neurons in blue, GPi neurons in red.

The mean firing rate of all the GPe and GPi recorded neurons during the baseline segment (B1) was 65.2±3.3 and 79.6±4.9 sp/s, respectively. The firing rates during movement (M) were 72.8±3.5 and 75.9±5 sp/s for the GPe and GPi, respectively (Fig. 3A). The mean firing rate, reflecting the total global activation, significantly increased during movement compared to B1 in the GPe (paired t-test, p<0.001), and remained unchanged in the GPi (paired t-test, p>0.05). The firing rates during the second baseline period (B2) were set back to their initial level, and were 65.9±3.4 and 78.4±5.3 for the GPe and GPi, respectively (paired t-test, p>0.05, relative to B1).

The response of the recorded neurons to movement, as depicted by limb acceleration, was characterized by a modulation of firing in a sinusoidal-like pattern, similar to the applied passive movement (Fig. 3B). mPETHs were constructed, and a sine function was fitted for each mPETH (Fig. 3C). A large majority of the neurons (81%, 54/67, in the GPe and 72%, 26/36, in the GPi) responded significantly (*R^2^*≥0.25 and *A*≥5) to movement of the upper limb and modulated their firing rate in relation to the movement cycles. A larger proportion of responding neurons was concentrated in the posterior areas of the GP (Fig. 2). The mean firing rate of movement-responding GPe cells was 73.5±3.7 and was significantly larger compared to the baseline firing rate (paired t-test, p<0.001). The mean firing rate for movement-responding GPi cells was 72.8±6 and was not significantly different from the baseline firing rate (paired t-test, p>0.05). The phase (*θ*), peak-to-mean amplitude (*A*) and the baseline rate (*r_0_*) parameters of the sine function were analyzed for the responding neurons. The phase (*θ*) was uniformly distributed (circular analysis test for non-uniformity, p>0.05) (Fig. 3D) and there was no spatial organization of the phases over the recording sites (Fig. 2). This wide distribution represents the diverse encoding of the movement cycle in the GP. The mean amplitude *(A)* of the modulation was 17.6±2 and 17.6±2.6 for the GPe and GPi cells respectively, and ranged from 5.77 (lower bound of 5) to 83.5 (Fig. 3D). The values of *r_0_* were highly correlated with the baseline firing rates (*r_0_* = 6.4+1.09*baselineRate, R^2^ = 0.76, p<0.001 for GPe cells, *r_0_* = 14.2+0.8*baselineRate, R^2^ = 0.72, p<0.001 for GPi cells, Fig. 3E). The amplitude of the response was not significantly correlated with *r_0_* (R^2^ = 0.04, p = 0.09). The phase was not significantly correlated with any of the other parameters (R^2^ = 0.02, p = 0.16 and R^2^ = 0.02, p = 0.21 for the correlation between cos(*θ*) to *r_0_* and *A*, respectively).

### Interaction analysis

**Figure 4:**
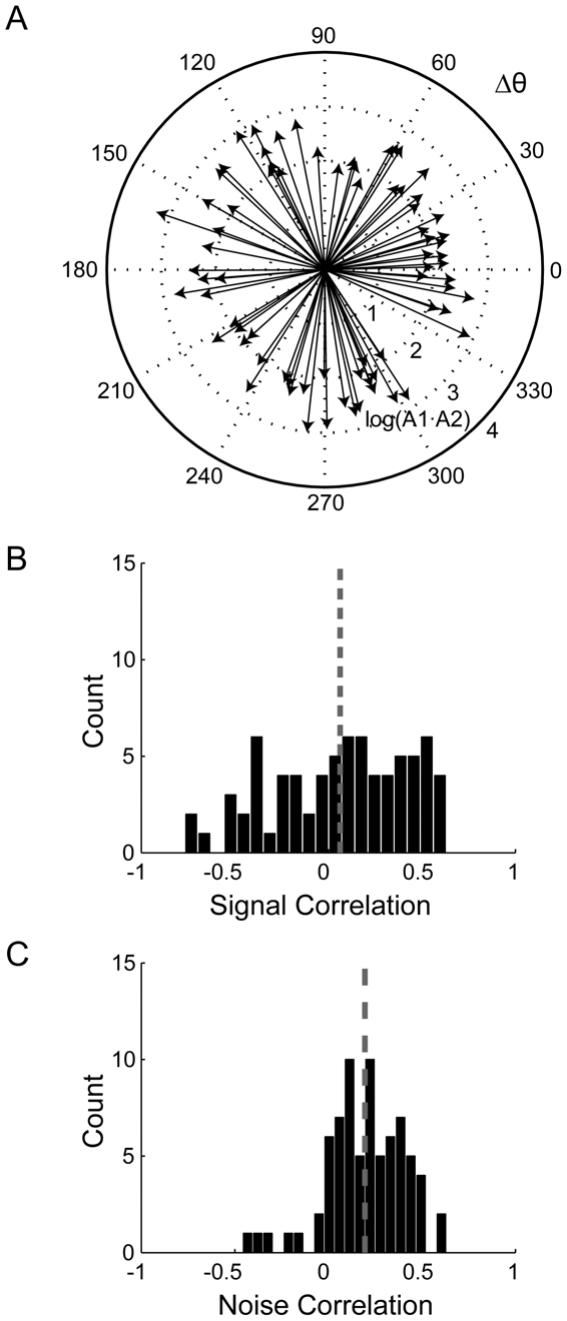
Interaction between pairs of pallidal neurons during movement. ***A***, Compass plot of interaction between responses to movement of all recorded pairs of neurons. Each arrow represents a pair of neurons: direction indicates the offset phase (Δθ) and length represents the multiplication of amplitudes of the sine fits (A1 and A2) on a logarithmic scale. ***B***, Histogram of signal correlation. ***C***, Histogram of noise correlation. Gray dashed lines in ***B*** and ***C*** indicate the mean value.

A total of 123 pairs of neurons were recorded simultaneously. No significant differences were found between pairs of neurons from the GPe and GPi, or mixed pairs; therefore all pairs were pooled together. In 74 pairs (60%) both cells responded significantly to movement and were included in the interaction analysis. The offset phase *(Δθ)* of all pairs of neurons was uniformly distributed (circular analysis test for non-uniformity, p>0.05) (Fig. 4A). This uniform distribution reflects the tendency of the neurons to fire independently of each other. Signal and noise correlations were calculated, differentiating the correlated activity of the neurons that could be related (“signal”) or could not be related (“noise”) to movement. The SC of all responding pairs was 0.06±0.04, and was not significantly different from 0 (t-test, p>0.05) (Fig. 4B), demonstrating that the neurons were not activated synchronously in response to movement. The SC values were diverse, as 42% (31/74) and 30% (22/74) of the pairs had a significant positive or negative correlation, respectively. The NC was 0.19±0.02, and was significantly larger than 0 (t-test, p<0.001) (Fig. 4C), implying that “background” activity, not related to movement, was partially synchronized. This was further supported by the evidence that 23% (17/74) of the pairs had a significant positive NC while only 2.7% (2/74) had a significant negative NC. The NC measures the inter-trial correlations, and thus demonstrates spontaneous co-activation in time scales of seconds.

## Discussion

In this study, we showed that a large majority of pallidal neurons in the parkinsonian state respond to cyclic passive limb movement of a single joint (elbow). The neuronal response to movement was sinusoidal-like and the phase of the peak response of the cells was uniformly distributed around the movement cycle. Simultaneously recorded neurons did not fire synchronously in relation to movement, but rather in a dispersed asynchronous pattern.

The baseline firing rates prior to the onset of movement in our study are consistent with previous studies that reported GPe and GPi firing rates in parkinsonian primates [24]–[26]. The high percentage of movement-responding neurons is in line with other studies that have found an increase in the probability of response to movement in the parkinsonian state compared to the normal state [10], [15], [16], [26], [27], though the percentages of responding neurons obtained in our study are even higher. This difference in the percentages of movement responses might be related to the larger number of movement repetitions used in our study and the stereotypic structure of the movement itself. The larger proportion of upper limb responding neurons in the posterior part of the GP found here is congruent with previous reports [4], [16].

This study reveals a dispersed encoding of movement, as demonstrated by: (1) the uniform distribution of the phase in the single neuron mPETHs; (2) the uniform distribution of the offset phase of simultaneously recorded neurons; and (3) the wide distribution of signal correlations.

The distribution of the mPETH phases, both temporally in relation to the movement cycle, and spatially over the GP area, demonstrates that the same movement is encoded differently by many neurons. This finding can be related to previous evidence concerning the receptive fields of pallidal neurons. It was reported that neurons in the parkinsonian state undergo a loss of specificity in response to movement. More pallidal cells were found to respond to movement compared to the normal state, usually in response to more than one joint [10], [26], [27]. Additionally, mixed phasic increases and decreases in discharge rate of GPi neurons were found following the application of elbow torque pulses [6], possibly indicating independent firing in response to movement. Another study reported loss of functional segregation downstream in the thalamus [11], where the neurons were nonspecific and correlated in the parkinsonian state. Our data extend these previous studies, and demonstrate that although many pallidal cells respond to the same movement, their response differs between cells. Despite careful performance of the movements, we cannot rule out the possibility that variation between movements might account for some of the variance between the neuronal responses evidenced in this study. However, even in the presence of such variability, this should affect the amplitude or the mean firing rate of the neuronal response, rather than the phase relative to the movement cycle, which was the prominent indicator of dispersed activity. The phase was not correlated with any of the other parameters of the response to movement, demonstrating that the properties of the phase are specific in themselves and are not a mere byproduct of any of the other quantities.

The dispersed response of the neurons can shed light on the encoding of movement by single neurons. However, the interaction between neurons, which is commonly used to explore and evaluate network connectivity, can only be studied using simultaneously recorded neurons. More specifically, the wide distribution of mPETH phases for single neurons is not necessarily related to the tendency of pairs of neurons to encode movement in a similar or dissimilar pattern. For example, despite a wide distribution of mPETH phases for all the population of single neurons, neurons that were recorded simultaneously could have had similar phases; in other words, they would tend to fire synchronously. This would result in a non-uniform distribution of offset phases, concentrated around zero. Another possibility is that the offset phases of simultaneously recorded neurons are randomly distributed, indicating that the neurons do not fire synchronously, but rather in a dispersed and independent manner. The uniform distribution of offset phases (Δθ) of pairs of neurons (Fig. 4A) reveals this dispersed nature of encoding of movement by showing that the difference between phases of single neurons are randomized and do not follow any organized pattern of activity.

We further analyzed the correlations and synchronization between pairs of neurons by using signal and noise correlations that discriminated correlated activity that resulted from common response to movement (SC) from spontaneous co-activation (NC), instead of using standard temporal cross-correlation analysis which is contaminated by the common response to movement. SC describes the correlation between neurons in the intra-cycle interval and quantifies common changes in the activity of the neurons in relation to movement. A maximal positive SC means that the peak responses of the two neurons tended to occur at the same phase of the movement cycle, whereas a maximal negative SC means that the peak responses tended to occur at a 180° phase difference. A 90° or 270° phase difference of peak responses results in zero SC, as the mPETHs of the two neurons are orthogonal. The “randomly” distributed movement-related locked discharge is reflected by the integration of three findings. First, the SC values are widely distributed, indicating that the pairs of neurons had different mPETH phases. Second, the number of pairs with significant correlations was high, with similar numbers of positive and negative correlations. Finally, the mean SC over all pairs was very close to zero. Altogether, these results indicate a dispersed encoding of movements at the network level.

The SC can be examined in relation to other studies that have addressed the synchrony issue. Previous studies have described correlated activity in the GP of parkinsonian primates [12] and in the subthalamic nucleus (STN) of PD patients [28]. However, this activity was limited to coherent oscillatory activity [17]. Correlated activity was found in spontaneous firing in the BG circuit and was attributed in some cases to tremor, but no direct encoding of the movement itself was described. The SC should also be distinguished from co-activation in response to movement, which measures the number of neurons that respond to the same movement, but not necessarily at the same time [10], [26], [27]. In our study, we directly explored the characteristics of the interaction between simultaneously recorded neurons in relation to movement and not only in relation to each other.

The complementary part of the SC is the NC, which considers the co-activation of pairs of neurons in the inter-cycle scale, thus neutralizing the common effect of movement. The NC extracts global common changes in activity in time scales of ∼1 second (duration of movement cycle), regardless of their oscillatory or non-oscillatory nature. Thus, the NC is different from previously reported correlations [12], [28] that primarily described oscillatory correlations at higher frequencies (>5 Hz). Unlike the SC, the NCs do not cancel each other out but rather are synergistic throughout the population. Therefore, even a small pairwise mean NC may potentially reflect a larger ensemble synchrony and might lead to a large modulation of the target.

In this study, passive movements were used due to the severe akinetic state of the parkinsonian animals. Therefore, it is reasonable to assume that the processed information was somatosensory and not purely motor. However, in either case the transmitted information was dispersed by the BG while conveying it back to the cortex. Additionally, both monkeys in this study exhibited dystonia, similar to other studies that used the same MPTP model for PD [24]. The dystonia was evident in the lower limbs and was not evident in the upper limbs during the recording sessions. However, we cannot rule out the possibility that some of the aspects of pallidal activity observed in this study may be related to dystonia. Furthermore, the characteristics of information transmission in the normal state remain unclear, as comparable data are unavailable. The dispersed encoding might be a property of normal activity as well, and further research and data are required to address this question.

The most widely held belief today is that neuronal activity in the basal ganglia is correlated and synchronized in the parkinsonian state. This has been previously demonstrated for spontaneous activity at rest [12], [17], [28]. Our findings complement these studies by observing the movement dependent activity and show for the first time that the activity in the GP in the parkinsonian state during movement is dispersed in nature, rather than synchronous. Further studies exploring activity in different nuclei of the BG circuit and involving multiple body parts are needed to provide a comprehensive picture of neuronal encoding during movement in the parkinsonian state and its abnormal characteristics.
